# Assembly and Comparative Analysis of the Complete Mitochondrial Genome of *Saussurea inversa* (Asteraceae)

**DOI:** 10.3390/genes15081074

**Published:** 2024-08-14

**Authors:** Wubin Dai, Xiuting Ju, Guomin Shi, Tao He

**Affiliations:** 1 School of Ecological Tourism, Sichuan University of Arts and Sciences, Dazhou 635000, China; daiwubin2024@163.com; 2 College of Agriculture and Animal Husbandry, Qinghai University, Xining 810016, China; 3 The Key Laboratory of Landscape Plants of Qinghai Province, Xining 810016, China; 4 State Key Laboratory of Plateau Ecology and Agriculture, Qinghai University, Xining 810016, China; 5 School of Eco-Environmental Engineering, Qinghai University, Xining 810016, China

**Keywords:** *Saussurea inversa*, mitochondrial genome, comparative analysis

## Abstract

*Saussurea inversa* is a perennial herb used in traditional Chinese medicine and is effective against rheumatoid arthritis. In this study, we sequenced the complete mitochondrial (mt) genome of *S. inversa* (GenBank accession number: ON584565.1). The circular mt genome of *S. inversa* was 335,372 bp in length, containing 62 genes, including 33 mRNAs, 22 tRNAs, 6 rRNAs, and 1 pseudogene, along with 1626 open reading frames. The GC content was 45.14%. Predictive analysis revealed substantial RNA editing, with *ccmFn* being the most abundantly edited gene, showing 36 sites. Gene migration between the mt and chloroplast (cp) genomes of *S. inversa* was observed through the detection of homologous gene fragments. Phylogenetic analysis revealed that *S. inversa* was clustered with *Arctium tomentosum* (Asteraceae). Our findings provide extensive information regarding the mt genome of *S. inversa* and help lay the foundation for future studies on its genetic variations, phylogeny, and breeding via the analysis of the mt genome.

## 1. Introduction

*S. inversa* commonly known as “Snow Lotus” in Tibetan medicine, is a perennial herb from the Asteraceae family [1], mainly found on the Qinghai–Tibetan Plateau. It grows in alpine limestone flats at altitudes of 4700–5400 m [2] and is a typical alpine plant well-adapted to the extreme environment [3]. It is effective against rheumatoid arthritis [4] and menstrual disorders when soaked in wine and then consumed; however, its specific mechanism of action needs further study. There is limited literature on *S. inversa*, particularly in genomics research [5]. In plants, mitochondrial genomes exhibit unique evolutionary patterns, they have a high rearrangement but a low mutation rate, as well as a large size [6]. They originate from a bacterial ancestor and maintain their own genome, which is expressed by designated mitochondrial transcription and translation machinery. This machinery differs from that used for nuclear gene expression, as the mitochondrial protein synthesis machinery is structurally and functionally very different from that governing eukaryotic cytosolic translation [7]. This study aims to analyze the complete mt genome of *S. inversa* to provide a scientific basis for understanding the adaptability of alpine plants to extreme environments and to help lay the foundation for future studies on the genetic variations, phylogeny, and breeding of *S. inversa.* It also provides theoretical support for the conservation and utilization of medicinal economic plants.

## 2. Materials and Methods

**Figure 1:**
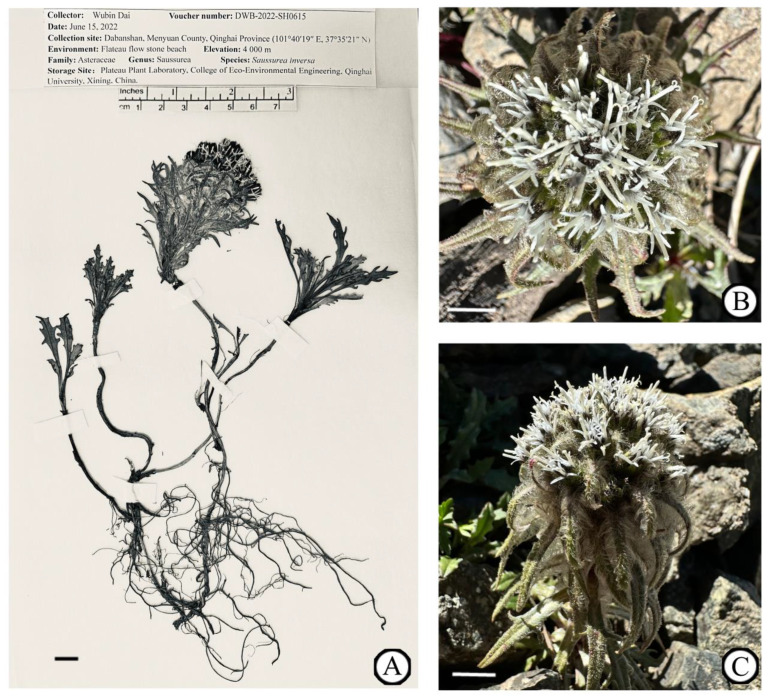
Reference image of *S. inversa*. (**A**) The specimen of *S. inversa* taken by Wubin Dai. (**B**) Inflorescence. (**C**) Arial part. Scale bar = 1 cm. (**B**,**C**) were taken by Wubin Dai at Daban Mountain, China (101°40′19″ E, 37°35′21″ N).

In this study, fresh leaves of *S. inversa* were collected from Daban Mountain (101°40′19″ E, 37°35′21″ N), a branch of the Qilian Mountains in the northeast of the Qinghai–Tibetan Plateau at an altitude of 4000 m. A specimen was deposited at the Plateau Plant Laboratory, College of Eco-Environmental Engineering, Qinghai University, Xining, China (Wubin Dai, daiwubin2024@163.com), under the voucher number DWB-2022-SH0615 (Figure 1).

The leaves were washed, frozen in liquid nitrogen, and ground to extract total DNA using the CTAB method. Second-generation sequencing, including sample quality detection, library construction, library quality detection, and the library sequencing process, was performed using the Illumina Novaseq 6000 platform (Illumina, Shanghai, China) according to the standard protocol provided by the manufacturer. The genomic DNA was randomly interrupted, and then the large fragments were enriched and purified using magnetic beads. Next, the large fragments were cut and recovered, and the fragmented DNA was repaired. After library construction, a certain concentration and volume of the DNA library was added to the flow cell, and the flow cell was transferred to the Oxford Nanopore PromethION sequencer for real-time single-molecule sequencing. The number of reads was 918,028, and the mean read length was 10,171. The resulting readable raw data were uploaded to GenBank, BioProject, and BioSample, with the SRA numbers PRJNA863745, SAMN30061603, and SRR20747203, respectively.

The sequencing data were assembled using Canu (https://canu.readthedocs.io/, accessed on 15 July 2022) following quality control. Annotations were performed for the mt genome using ORFF (www.ncbi.nlm.nih.gov/orffinder, accessed on 15 July 2022), for tRNA using tRNAscanSE [8], and for the encoding protein and rRNA using BLAST (blast.ncbi.nlm.nih.gov). RNA editing sites were predicted using PREP Suite [9]. An mt genome map of *S. inversa* and the structures of the genes that were difficult to annotate was prepared and analyzed using OGVIEW [10] (http://www.1kmpg.cn/pmgmap, accessed on 15 July 2022).

Conserved CDSs (coding sequences) of eight Asteraceae, two Rosaceae, and one Leguminosae (outgroup) were selected to draw a maximum likelihood evolutionary tree. These sequences were compared with multiple sequences using MAFFT software (v7.427, Auto mode), and the compared sequences were joined head to tail and then trimmed with trimAl (v1.4.rev15). The model prediction was carried out using Jmodeltest-2.1.10 software after trimming to confirm that the model was of the GTR type. The bootstrap test was performed 1000 times, and support values, shown as percentages, are shown at the nodes [11].

## 3. Results

**Figure 2:**
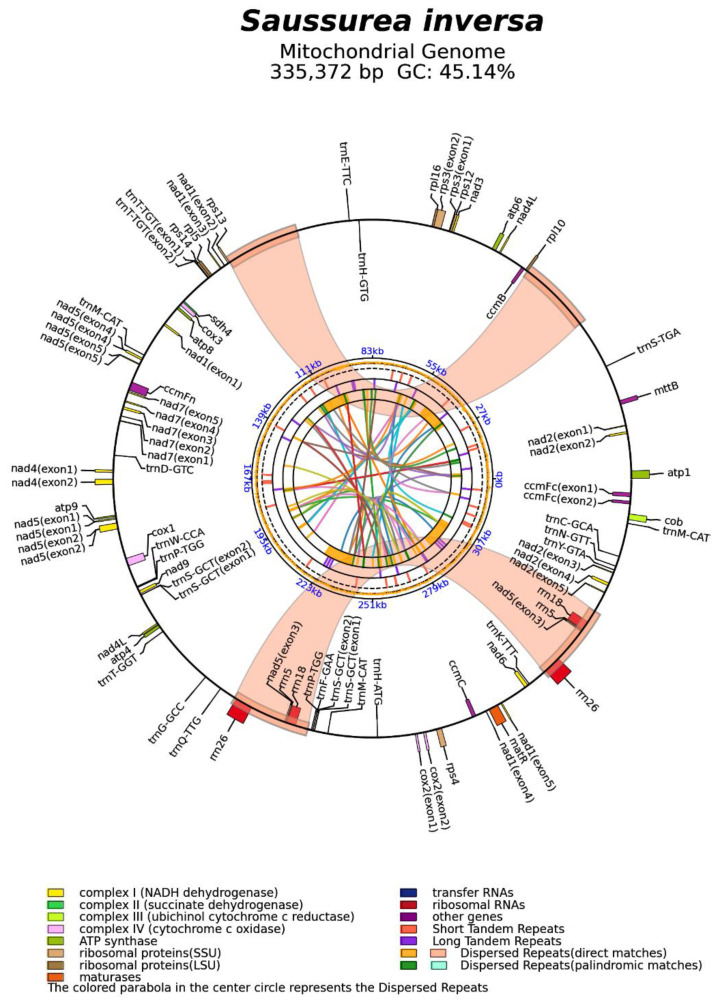
Mt genome map of *S. inversa*. The forward coding gene is on the outside of the circle, and the reverse coding gene is inside the circle. The colored parabola in the center of the circle represents the dispersed repeats.

Genomic features of the *S. inversa* mt genome. The minimum, maximum, and average depths of coverage of the assembled mt genome were 9, 4227, and 76, respectively (Figure S1). The mt genome map (Figure 2) showed that the circular mt genome of *S. inversa* was 335,372 bp in length. There were 1626 open reading frames and 62 known genes, including 22 tRNAs, 6 rRNAs, and 1 pseudogene. Among the 62 known genes, 33 mRNAs were included, namely ATP synthases (*atp1*, *atp4*, *atp6*, *atp8*, and *atp9*), cytochrome c biogenesis (*ccmB*, *ccmC*, *ccmFc*, and *ccmFn*), ubiquinol cytochrome c reductase (*cob*), cytochrome c oxidase (*cox1*, *cox2*, and *cox3*), maturase (*matR*), transport membrane protein (*mttB*), NADH dehydrogenase (*nad1*, *nad2*, *nad3*, *nad4*, *nad4L*, *nad5*, *nad6*, *nad7*, and *nad9*), ribosomal proteins (LSU:*rpl10*, *rpl16*, *rpl5*; SSU:*rps12*, *rps13*, *rps3*, and *rps4*), and succinate dehydrogenase (*sdh4*). It is worth noting that both *nad4L* and *nad5* had two copies, and eight genes contained introns (ccmFc*, cox2*, nad1****, nad2****, nad4*, nad5****, nad7****, and rps3*, where * represents the number of introns). There were seven cis-splicing genes, which were *ccmFc*, *cox2*, *nad4*, *nad7*, *rps3*, *trnS-GCT*, and *trnT-TGT*, and three trans-splicing genes, which were *nad1*, *nad2*, and *nad5*.

**Figure 3:**
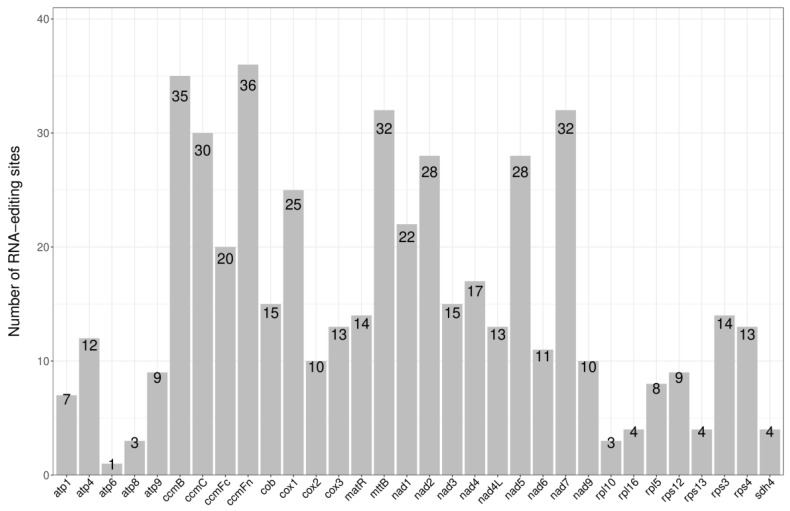
The number of RNA editing sites.

Prediction of RNA editing sites. RNA editing is a generic term comprising a variety of processes that alter the DNA-encoded sequence of a transcribed RNA by inserting, deleting, or modifying nucleotides in the transcripts [9]. For instance, restoration of the start codon of the *atp6* gene from ACG to ATG in Table 1 requires RNA editing. There are large amounts of RNA editing for the genes listed above, and the number of RNA edits for each gene is shown in Figure 3. The codon position, change of amino acids, and type of RNA editing are listed in Supplementary Table S1. The highest number of RNA edits was for *ccmFn*, with 36, and the lowest was for *atp6*, with only one. After RNA editing, 44.67% of the amino acids retained their hydrophobicity, while 8.05% of hydrophobic amino acids became hydrophilic, and 47.28% of hydrophilic amino acids became hydrophobic (Table 2).

**Figure 4:**
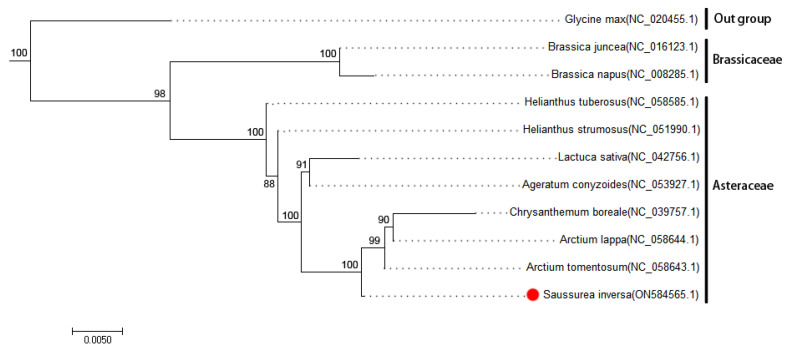
Phylogenetic tree of *S. inversa*. The following sequences were used to establish the phylogenetic tree: *G. max* NC_020455.1 [12]; *B. juncea* NC_016123.1 [13]; *B. napus* NC_008285.1 [14]; *H. tuberosus* NC_058585.1, *H. strumous* NC_051990.1, *L. sativa* NC_042756.1, and *A. conyzoides* NC_053927.1 [15]; *C. boreale* NC_039757.1, *A. lappa* NC_058644.1, and *A. tomentosum* NC_058643.1. Among them, *G. max* as an outgroup and *B. juncea* and *B. napus* as two species of Brassicaceae clustered together into a separate group. The GenBank accession numbers of each species are shown in parentheses. Bootstrap support values in percentages are shown at the nodes.

Phylogenetic analysis. To understand the process of evolution of the *S. inversa* mt genome, we conducted a phylogenetic analysis of the *S. inversa* mt genome and the published mt genomes of 12 plants. The clustering in the phylogenetic tree is consistent with the relationships of these species at the family and genus levels, indicating that the mt genome-based clustering results are reliable. The phylogenetic tree (Figure 4) revealed that *S. inversa* is closely related to *A. tomentosum* (Asteraceae). The reliability of the support values of each node was above 88%, indicating that the evolutionary tree accurately reflects the genetic distances between the listed species.

**Figure 5:**
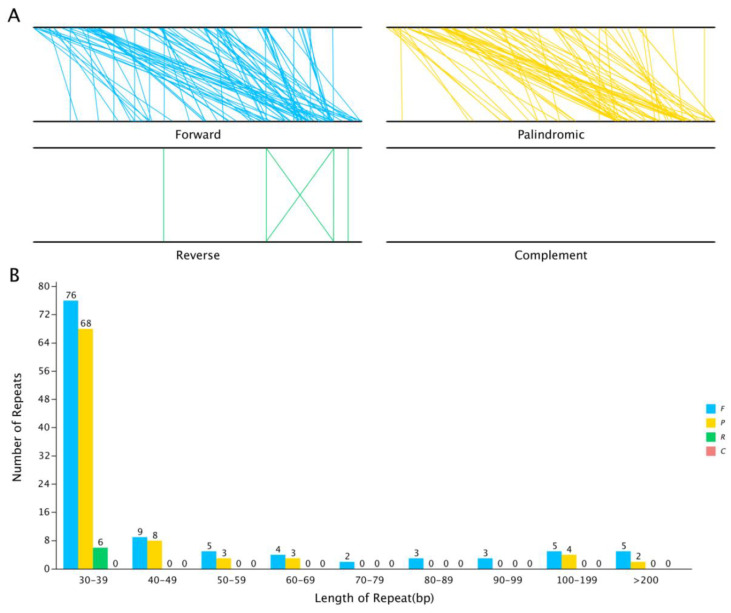
The interspersed repeat sequences in the *S. inversa* mt genome. (**A**) The four interspersed repeat types are distributed throughout the genome; the two black lines represent the mt genome, and the same repeats are associated with the line segments. (**B**) Distribution of the lengths of the interspersed repeats in the mt genome. The abscissa indicates the type of interspersed repeat, and the ordinate indicates the number of scattered repeats. F for Forward, P for Palindromic, R for Reverse, and C for Complement.

Repeat sequence analysis. Interspersed repeat sequences are repetitive sequences that are scattered in the genome. In the *S. inversa* mt genome, we identified a total of 206 interspersed repeats with a length greater than or equal to 30 bp; of these, 112 were forward repeats and 88 were palindrome repeats. The length of the longest forward repeat sequence was 16,421 bp, and that of the longest palindrome repeat sequence was 518 bp. The distribution of the lengths of the forward and palindrome repeats is shown in Figure 5. The abundance of both types of repeats was the highest when the repeats were in the range of 30–39 bp.

**Figure 6:**
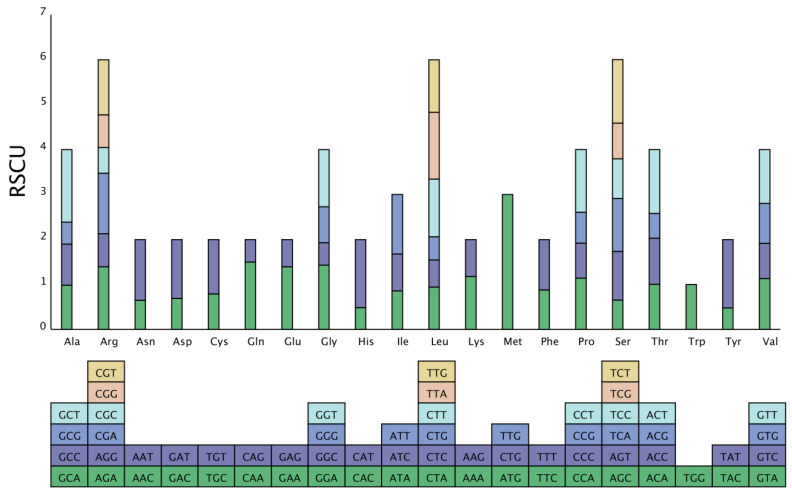
RSCU in the *S. inversa* mt genome. The different amino acids are shown on the *x*-axis. RSCU values are the number of times a particular codon was observed relative to the number of times that codon would be expected for uniform synonymous codon usage.

Analysis of the codon composition. Due to codon concatenation, each amino acid corresponds to a minimum of one codon and a maximum of six codons. There is great variation in the rate of codon usage in the genomes of different species and organisms. This inequality in synonymous codon usage is called relative synonymous codon usage (RSCU). This preference is believed to be the combined result of natural selection, species mutation, and genetic drift [16]. We used a self-coded Perl script to analyze the codon composition of the *S. inversa* mt genome. The results are shown in Table 3. The number of codons in all coding genes was 9881, and the relative RSCU in the *S. inversa* mt genome is shown in Figure 6. There were 31 codons with an RSCU > 1, indicating that the usage frequency of these codons is greater than that of other synonymous codons. Among these, 28 codons ending with the A/T base were identified, accounting for 90.32% of the codons, indicating that frequently used codons tend to end with the A/T base.

**Figure 7:**
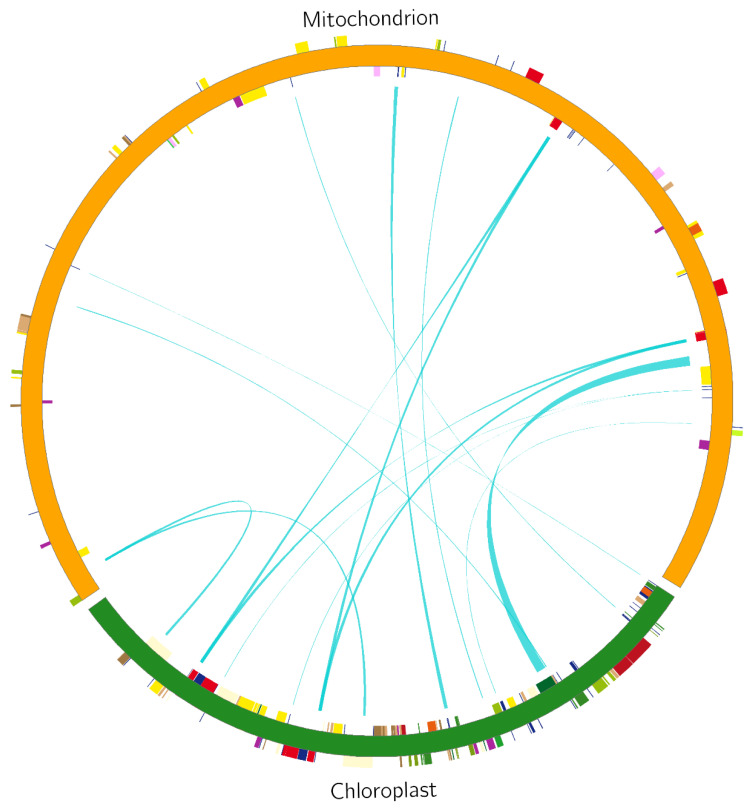
Comparison of a homologous fragment in the *S. inversa* cp genome (GenBank accession number: MT554929) to that in the mt genome. The green line segment of the circle represents the *S. inversa* cp genome, and the red line segment represents the *S. inversa* mt genome. Genes from the same complex are labelled with the same colours. The green line segment in the circle connects the start and end points of the transferred gene fragments. The width of the green line segment represents the size of the transferred fragment.

Analysis of homologous fragments of mitochondria and chloroplasts. Using the BLAST tool, we screened the fragments of the *S. inversa* mt and cp genomes [5] exhibiting > 70% similarity and performed homologous fragment analysis (Figure 7). We screened out 15 homologous fragments with a total length of 9078 bp, which accounted for 2.70% of the mt genome (Table 3). These homologous fragments contained 14 annotated genes, of which six were tRNA genes, namely *tRNA-CCA*, *tRNA-UGG*, *tRNA-GUC*, *tRNA-GUG*, *tRNA-GUU*, and *tRNA-CAU*, while the others were *rrn16*, *psaB*, *psaA*, *ycf2*, *petL*, *petG*, *rbcL*, and *rps14*.

## 4. Discussion

Mitochondria have a relatively independent genetic transcription system [17]. However, the mt genome contains not only its genes but also some genes from the cp genome, and they have multiple types of repetitive sequences and relatively conserved coding sequences. This suggests that these genes may have migrated during evolution, and further investigation is needed to understand the specific mechanism. Rapid advances in genome sequencing technology have accelerated the study of the mt genome. This study described the basic features of the mt genome of *S. inversa* for the first time, which provides an important foundation for understanding the function, inheritance, and evolutionary trajectory of the mt genome. The mt genome of *S. inversa* is a circular sequence with a length of 335,372 bp and a GC content of 45.14%. Plants exhibit large mitochondrial genomes of 66 Kb to 11.3 Mb, with large intergenic repetitions prone to recombination [18]. The size of the mitochondrial genome varies considerably; despite this, the GC content is relatively conservative. For example, the GC content was found to be 45.08% for watermelon (*Citrullus lanatus* [Thunb.] Matsum. Et Nakai) and 44.77% for melon (*Cucumis melo* L.) [19]. which is similar to that of *S. inversa*. We performed BLAST analysis of the mt sequence and annotated the sequence using software, and we found 33 protein-coding genes, 22 tRNA genes, 4 rRNA genes, and 1626 ORFs in the mt genome. Since sequence duplications can lead to intermolecular recombination in mitochondria, it is particularly important to perform repetitive sequence analysis, and we analyzed the dispersed repetitive sequences of the mt genome of *S. inversa*. The results showed a total of 206 dispersed repeat sequences with lengths greater than or equal to 30 bp. The length of the longest forward repeat sequence was 16,421 bp, and that of the longest palindrome repeat sequence was 518 bp.

RNA editing is a post-transcriptional modification process that alters the RNA sequence relative to the genomic blueprint. In plant mitochondria, the most common type is C-to-U, and the absence of C-to-U RNA editing results in abnormal plant development, such as etiolation and albino leaves, aborted embryonic development, and retarded seedling growth [20]. In this study, 497 RNA editing sites were identified in 34 coding genes of the *S. inversa* genome, with 29 codon shift types. Among the codon transfer types, TCA→TTA was the most common, which is consistent with the findings of Qiao et al. [21], who noted 69 editing sites. After RNA editing, 8.12% of hydrophobic amino acids became hydrophilic, and 47.28% of hydrophilic amino acids became hydrophobic. Consistent results exist for the *Bupleurum chinense* DC. mt genome, where the most abundant transfer type in the plant was TCA→TTA, totaling 78, which was edited to change the hydrophobicity of more than half of the amino acids [21]. The selection of editing sites in the genome of *S. inversa* was strongly biased, with all editing sites being C-to-U edited, which is the most common type of editing in plant mt genomes [20]. It was shown that RNA editing occurring at the second position of the codon accounted for more than half of the total number of edits [22]. In the *S. inversa* mt genome, 78.87% of the editing sites were also located at the second base of the triplet codon, which is consistent with previous findings. In addition, after RNA editing, the encoded amino acids were partially converted into termination codons [22]. In the mt genome of *S. inversa*, 0.80% of the amino acids were edited into termination codons, resulting in an early stop of the coding process, which altered the gene’s function.

Plant DNA transfer between organellar and nuclear genomes and between species occurs frequently, and sequencing analyses have identified many DNA transfer events between different plant genomes (mitochondrial, nuclear, and chloroplast). It was found that DNA transfer events primarily involve the transfer of DNA fragments from the organelle genome to the nuclear genome, followed by the transfer of nuclear and plastid genomes to the mt genome [19]. In the present study, we found that the total length of homologous fragments transferred from the cp genome to the mt genome of *S. inversa* was 9078 bp, and these homologous fragments contained eight annotated genes, six of which were tRNA genes. This result is similar to that of Ma et al. [23], who found that the fragment of the *Acer truncatum* cp genome transferred to the mt genome contained six integrative genes, five of which were tRNA genes. The percentage of transferred fragments in the mt genome of *S. inversa* was 2.7%, which is similar to the data previously reported for *A. truncatum* (2.36%) [23] and *Salix suchowensis* (2.8%) [24], but lower than that of *Suaeda glauca* (5.18%) [25]. In melon mitochondria, 48.62% of the sequence genome was homologous to the nuclear genome, and 1048 fragments in the mitochondria corresponded to 3391 fragments on the nuclear genome. The 1048 homologous sequences in the mitochondrial genome ranged from 214 to 6120 bp, with an average length of 941 bp [19]. However, the nuclear genes of *S. inversa* are not yet publicly available, so subsequent analyses will be carried out at a later time.

We analyzed the codons in the *S. inversa* mt genome, and the RSCU value reflects the ratio of the actual frequency of use of a codon to the theoretical frequency of use in the absence of usage bias; if RSCU = 1, it means that there is no bias in the use of the codon, and if RSCU < 1, it means that the actual frequency of use of the codon is lower than that of the other synonymous codons. Conversely, if RSCU > 1, it means that the actual frequency of use of the codon is higher than the usage frequency of other synonymous codons [26]. The results of the analysis showed that there were 31 codons with an RSCU > 1, indicating that the usage frequency of these codons is greater than that of other synonymous codons. Among these, 28 codons ending with the A/T base were identified, accounting for 90.32% of the codons, indicating that frequently used codons tend to end with the A/T base.

## 5. Conclusions

In this study, the mt genome of *S. inversa* was sequenced, assembled, and annotated, and the DNA and amino acid sequences of the annotated genes were analyzed. The mt genome of *S. inversa* is circular and has a total length of 335,372 bp. A total of 62 genes were annotated in the mt genome, including 33 protein-coding genes, 22 tRNA genes, and 6 rRNA genes. We analyzed repeat sequences, RNA editing processes, and codon preferences in the mt genome of *S. inversa*. We observed gene transfer between the mt and cp genomes of *S. inversa* by examining homologous fragments. In addition, our results showed that although the size of plant mt genomes varies greatly, their GC content is relatively conserved during evolution, suggesting that the mt gene is conserved during evolution. This study provides extensive information about the mt genome of *S. inversa*. Importantly, this study lays the foundation for future studies on the genetic variation, phylogeny, and plateau adaptation of *S. inversa* using the mt genome.

## Figures and Tables

**Table 1:** Basic information of the *S. inversa* mt genome genes.

Group of Genes	Gene Name	Length	Start Codon	Stop Codon	Amino Acid
ATP synthase	*atp1*	1533	ATG	TAA	511
	*atp4*	576	ATG	TAA	192
	*atp6*	774	ACG(ATG)	TAA	258
	*atp8*	480	ATG	TAA	160
	*atp9*	261	ATG	TAA	87
Cytochrome c biogenesis	*ccmB*	621	ATG	TGA	207
	*ccmC*	753	ATG	TGA	251
	*ccmFc*	1320	ATG	TAA	440
	*ccmFn*	1719	ATG	TAG	573
Ubiquinol cytochrome c reductase	*cob*	1176	ATG	TGA	392
Cytochrome c oxidase	*cox1*	1584	ATG	TAA	528
	*cox2*	834	ATG	TAG	278
	*cox3*	798	ATG	TGA	266
Maturase	*matR*	1968	ATG	TAG	656
Transport membrane protein	*mttB*	801	ATT	TAG	267
NADH dehydrogenase	*nad1*	978	ATG	TAA	326
	*nad2*	1467	ATG	TAA	489
	*nad3*	357	ATG	TAA	119
	*nad4*	1488	ATG	TGA	496
	*nad4L*	273	ATG	TAA	91
	*nad4L*	273	ATG	TAA	91
	*nad5*	2013	ATG	TAA	671
	*nad5*	2013	ATG	TAA	671
	*nad6*	729	ATG	TAA	243
	*nad7*	1185	ATG	TAG	395
	*nad9*	573	ATG	TAA	191
Ribosomal proteins (LSU)	*rpl10*	489	ATG	TAA	163
	*rpl16*	423	ATG	TGA	141
	*rpl5*	564	ATG	TAA	188
Ribosomal proteins (SSU)	*rps12*	378	ATG	TGA	126
	*rps13*	351	ATG	TGA	117
	*rps3*	1710	ATG	TAG	570
	*rps4*	1086	ATG	TAG	362
Succinate dehydrogenase	*sdh4*	387	ATG	CGA(TGA)	129
Ribosomal RNAs	*rrn18*	1937			
	*rrn18*	1937			
	*rrn26*	3619			
	*rrn26*	3619			
	*rrn5*	117			
	*rrn5*	117			
Transfer RNAs	*trnC-GCA*	71			
	*trnD-GTC*	74			
	*trnE-TTC*	72			
	*trnF-GAA*	74			
	*trnG-GCC*	73			
	*trnH-ATG*	69			
	*trnH-GTG*	74			
	*trnK-TTT*	73			
	*trnM-CAT*	73			
	*trnM-CAT*	74			
	*trnM-CAT*	73			
	*trnN-GTT*	72			
	*trnP-TGG*	74			
	*trnP-TGG*	75			
	*trnQ-TTG*	72			
	*trnS-GCT*	71			
	*trnS-GCT*	71			
	*trnS-TGA*	87			
	*trnT-GGT*	74			
	*trnT-TGT*	73			
	*trnW-CCA*	74			
	*trnY-GTA*	83			

**Table 2:** Prediction of RNA editing sites.

Type	RNA Editing	Number	Percentage
Hydrophilic–hydrophilic	CAC (H)→TAC (Y)	6	
	CAT (H)→TAT (Y)	17	
	CGC (R)→TGC (C)	10	
	CGT (R)→TGT (C)	28	
	total	61	12.27%
Hydrophilic–hydrophobic	ACA (T)→ATA (I)	3	
	ACC (T)→ATC (I)	2	
	ACG (T)→ATG (M)	5	
	ACT (T)→ATT (I)	4	
	CGG (R)→TGG (W)	33	
	TCA (S)→TTA (L)	69	
	TCC (S)→TTC (F)	27	
	TCG (S)→TTG (L)	40	
	TCT (S)→TTT (F)	52	
	total	235	47.28%
Hydrophilic–stop	CAG (Q)→TAG (X)	2	
	CGA (R)→TGA (X)	2	
	total	4	0.80%
Hydrophobic–hydrophilic	CCA (P)→TCA (S)	7	
	CCC (P)→TCC (S)	11	
	CCG (P)→TCG (S)	6	
	CCT (P)→TCT (S)	16	
	total	40	8.05%
Hydrophobic–hydrophobic	CCA (P)→CTA (L)	49	
	CCC (P)→CTC (L)	7	
	CCC (P)→TTC (F)	6	
	CCG (P)→CTG (L)	36	
	CCT (P)→CTT (L)	19	
	CCT (P)→TTT (F)	12	
	CTC (L)→TTC (F)	6	
	CTT (L)→TTT (F)	15	
	GCG (A)→GTG (V)	4	
	GCT (A)→GTT (V)	3	
	total	157	31.59%
	All	497	100%

**Table 3:** Comparison of a homologous fragment in the *S. inversa* cp genome to that in the mt genome.

Matches (%)	Length	Cap	cp Start	cp End	mt Start	mt End	Genes
99.922	2556	1	36,401	38,955	307,643	310,198	*psaB* (partial: 98.64%); *psaA* (partial: 15.76%)
91.348	601	5	87,770	88,358	5938	5347	*ycf2* (partial: 8.57%)
91.348	601	5	147,195	147,783	5347	5938	*ycf2* (partial: 8.57%)
80.285	913	40	65,237	66,122	190,138	191,003	*petL*; *petG*; *tRNA-CCA*; *tRNA-UGG*
73.874	888	39	99,807	100,670	235,198	234,340	*rrn16* (partial: 57.99%)
73.874	888	39	134,883	135,746	234,340	235,198	*rrn16* (partial: 57.99%)
73.874	888	39	99,807	100,670	303,750	302,892	*rrn16* (partial: 57.99%)
73.874	888	39	134,883	135,746	302,892	303,750	*rrn16* (partial: 57.99%)
93.939	198	3	55,269	55,461	207,773	207,576	*rbcL* (partial: 13.52%)
89.163	203	0	35,948	36,150	77,007	76,805	*rps14* (partial: 61.52%)
90.476	126	1	11,104	11,226	162,819	162,944	*tRNA-GUC*
97.531	81	0	2	82	86,537	86,617	*tRNA-GUG*
94.048	84	1	127,807	127,889	316,935	316,852	*tRNA-GUU*
94.048	84	1	107,664	107,746	316,852	316,935	*tRNA-GUU*
94.937	79	0	51,506	51,584	326,025	326,103	*tRNA-CAU*

## Data Availability

The data presented in this study are openly available in NCBI at https://www.ncbi.nlm.nih.gov/nuccore/ON584565.1 (accessed on 24 May 2022), GenBank accession number ON584565.1.

## Supplementary Materials

The following supporting information can be downloaded at: https://www.mdpi.com/article/10.3390/genes15081074/s1, Figure S1: Statistical depth map. Raw second-generation sequencing reads were aligned to the assembled genomes using bortie2 (2.3.5.1) and then sorted using Samtools (1.9) with statistical depth; Table S1: Codon position, change of amino acids, and type of RNA editing information.
