# Supplementary material for: Assembly and Comparative Analysis of the Complete Mitochondrial Genome of Saussurea inversa (Asteraceae)
Source: Genes (Basel). 2024 Aug 14;15(8):1074. doi: 10.3390/genes15081074 (PMC11353396; doi:10.3390/genes15081074)
Supplement: Supplementary file 1 [file genes-15-01074-s001.zip › genes-3129405-supplementary.pdf]

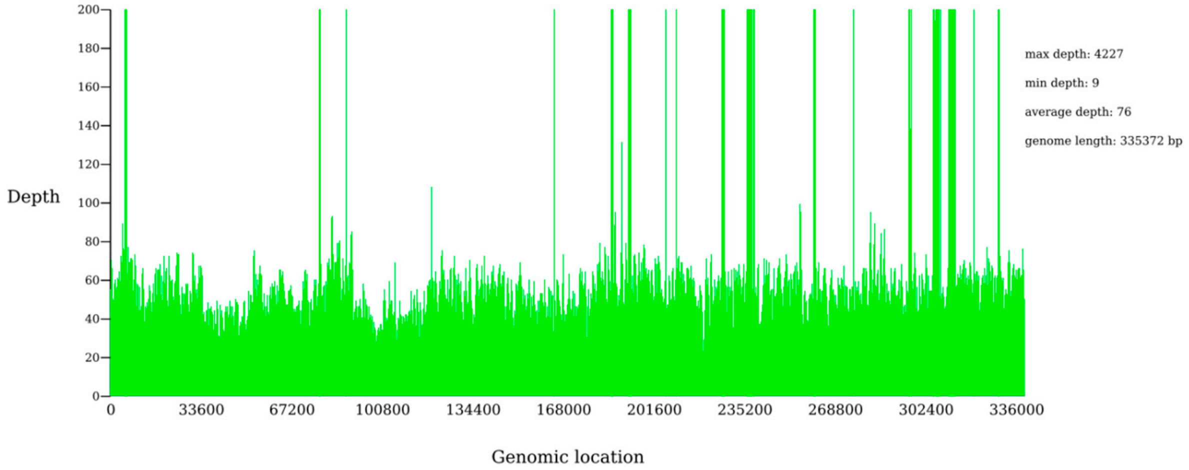

Figure S1 Statistical depth map. Raw second-generation sequencing reads were aligned to the assembled genomes using bortie2 (2.3.5.1) and then sorted using Samtools (1.9) with statistical depth.

Supplementary Table S1 Codon position, change of amino acids, and type of RNA editing infomation

Gene	Nucleotide position	Amino acid position	Type of RNA editing
atp1	1039	347	CCT (P) => TCT (S)
atp1	1064	355	TCG (S) => TTG (L)
atp1	1178	393	TCA (S) => TTA (L)
atp1	1216	406	CTT (L) => TTT (F)
atp1	1292	431	CCG (P) => CTG (L)
atp1	1490	497	CCA (P) => CTA (L)
atp1	1526	509	ACA (T) => ATA (I)
atp4	59	20	TCT (S) => TTT (F)
atp4	71	24	TCA (S) => TTA (L)
atp4	89	30	TCA (S) => TTA (L)
atp4	118	40	CGT (R) => TGT (C)
atp4	215	72	TCA (S) => TTA (L)
atp4	226	76	CTC (L) => TTC (F)
atp4	248	83	CCT (P) => CTT (L)
atp4	251	84	CCG (P) => CTG (L)
atp4	395	132	TCA (S) => TTA (L)
atp4	407	136	CCA (P) => CTA (L)
atp4	416	139	ACT (T) => ATT (I)
atp4	490	164	CCC (P) => TCC (S)
atp6	8	3	ACG (T) => ATG (M)

atp8	47	16	TCA (S) => TTA (L)
atp8	58	20	CTC (L) => TTC (F)
atp8	452	151	CCA (P) => CTA (L)
atp9	50	17	TCA (S) => TTA (L)
atp9	82	28	CTT (L) => TTT (F)
atp9	92	31	TCG (S) => TTG (L)
atp9	134	45	TCA (S) => TTA (L)
atp9	182	61	TCG (S) => TTG (L)
atp9	191	64	CCA (P) => CTA (L)
atp9	212	71	TCA (S) => TTA (L)
atp9	215	72	TCC (S) => TTC (F)
atp9	223	75	CGA (R) => TGA (X)
ccmB	38	13	ACC (T) => ATC (I)
ccmB	43	15	CCC (P) => TCC (S)
ccmB	71	24	CCA (P) => CTA (L)
ccmB	80	27	TCA (S) => TTA (L)
ccmB	137	46	TCC (S) => TTC (F)
ccmB	149	50	CCG (P) => CTG (L)
ccmB	154	52	CGG (R) => TGG (W)
ccmB	160	54	CCC (P) => TCC (S)
ccmB	164	55	CCG (P) => CTG (L)
ccmB	172	58	CCT (P) => TCT (S)
ccmB	179	60	CCT (P) => CTT (L)
ccmB	193	65	CCT (P) => TTT (F)
ccmB	194	65	CCT (P) => TTT (F)
ccmB	286	96	CGG (R) => TGG (W)
ccmB	304	102	CGT (R) => TGT (C)
ccmB	313	105	CGT (R) => TGT (C)
ccmB	338	113	CCG (P) => CTG (L)
ccmB	367	123	CGG (R) => TGG (W)
ccmB	380	127	CCA (P) => CTA (L)
ccmB	424	142	CGT (R) => TGT (C)
ccmB	428	143	TCG (S) => TTG (L)
ccmB	467	156	TCG (S) => TTG (L)
ccmB	476	159	CCA (P) => CTA (L)
ccmB	485	162	TCA (S) => TTA (L)
ccmB	494	165	TCA (S) => TTA (L)
ccmB	503	168	CCA (P) => CTA (L)
ccmB	512	171	TCT (S) => TTT (F)
ccmB	514	172	CGT (R) => TGT (C)
ccmB	551	184	TCA (S) => TTA (L)
ccmB	554	185	TCG (S) => TTG (L)
ccmB	566	189	TCC (S) => TTC (F)
ccmB	572	191	CCG (P) => CTG (L)
ccmB	587	196	TCT (S) => TTT (F)
ccmB	596	199	TCG (S) => TTG (L)
ccmB	611	204	TCA (S) => TTA (L)
ccmC	76	26	CGG (R) => TGG (W)
ccmC	103	35	CAT (H) => TAT (Y)

ccmC	115	39	CGG (R) => TGG (W)
ccmC	133	45	CTT (L) => TTT (F)
ccmC	161	54	CCT (P) => CTT (L)
ccmC	179	60	GCG (A) => GTG (V)
ccmC	184	62	CGG (R) => TGG (W)
ccmC	281	94	ACA (T) => ATA (I)
ccmC	299	100	TCT (S) => TTT (F)
ccmC	331	111	CGG (R) => TGG (W)
ccmC	395	132	TCG (S) => TTG (L)
ccmC	400	134	CTT (L) => TTT (F)
ccmC	421	141	CGT (R) => TGT (C)
ccmC	436	146	CCT (P) => TCT (S)
ccmC	446	149	CCG (P) => CTG (L)
ccmC	451	151	CCT (P) => TCT (S)
ccmC	458	153	TCA (S) => TTA (L)
ccmC	463	155	CGT (R) => TGT (C)
ccmC	467	156	GCT (A) => GTT (V)
ccmC	473	158	CCG (P) => CTG (L)
ccmC	497	166	TCT (S) => TTT (F)
ccmC	521	174	TCG (S) => TTG (L)
ccmC	568	190	CCT (P) => TCT (S)
ccmC	575	192	CCC (P) => CTC (L)
ccmC	605	202	TCC (S) => TTC (F)
ccmC	608	203	CCC (P) => CTC (L)
ccmC	614	205	TCA (S) => TTA (L)
ccmC	619	207	CGT (R) => TGT (C)
ccmC	656	219	CCA (P) => CTA (L)
ccmC	673	225	CCT (P) => TCT (S)
ccmFc	38	13	TCC (S) => TTC (F)
ccmFc	50	17	CCT (P) => CTT (L)
ccmFc	52	18	CGT (R) => TGT (C)
ccmFc	103	35	CCC (P) => TCC (S)
ccmFc	119	40	TCT (S) => TTT (F)
ccmFc	122	41	TCT (S) => TTT (F)
ccmFc	151	51	CCT (P) => TCT (S)
ccmFc	155	52	TCA (S) => TTA (L)
ccmFc	160	54	CCT (P) => TCT (S)
ccmFc	203	68	ACG (T) => ATG (M)
ccmFc	310	104	CGT (R) => TGT (C)
ccmFc	391	131	CGT (R) => TGT (C)
ccmFc	406	136	CGT (R) => TGT (C)
ccmFc	620	207	GCG (A) => GTG (V)
ccmFc	704	235	GCT (A) => GTT (V)
ccmFc	878	293	TCT (S) => TTT (F)
ccmFc	1139	380	CCA (P) => CTA (L)
ccmFc	1160	387	CCG (P) => CTG (L)
ccmFc	1234	412	CGG (R) => TGG (W)
ccmFc	1315	439	CGA (R) => TGA (X)
ccmFn	8	3	ACG (T) => ATG (M)

ccmFn	44	15	CCG (P) => CTG (L)
ccmFn	104	35	CCT (P) => CTT (L)
ccmFn	143	48	CCG (P) => CTG (L)
ccmFn	148	50	CGT (R) => TGT (C)
ccmFn	157	53	CCT (P) => TCT (S)
ccmFn	254	85	TCA (S) => TTA (L)
ccmFn	262	88	CGG (R) => TGG (W)
ccmFn	289	97	CTT (L) => TTT (F)
ccmFn	340	114	CAT (H) => TAT (Y)
ccmFn	362	121	TCC (S) => TTC (F)
ccmFn	371	124	TCG (S) => TTG (L)
ccmFn	484	162	CGT (R) => TGT (C)
ccmFn	556	186	CCT (P) => TTT (F)
ccmFn	557	186	CCT (P) => TTT (F)
ccmFn	703	235	CCT (P) => TTT (F)
ccmFn	704	235	CCT (P) => TTT (F)
ccmFn	713	238	TCG (S) => TTG (L)
ccmFn	751	251	CGT (R) => TGT (C)
ccmFn	773	258	TCA (S) => TTA (L)
ccmFn	785	262	CCA (P) => CTA (L)
ccmFn	800	267	TCA (S) => TTA (L)
ccmFn	949	317	CGC (R) => TGC (C)
ccmFn	1264	422	CGG (R) => TGG (W)
ccmFn	1292	431	CCA (P) => CTA (L)
ccmFn	1309	437	CAT (H) => TAT (Y)
ccmFn	1324	442	CGG (R) => TGG (W)
ccmFn	1342	448	CGG (R) => TGG (W)
ccmFn	1375	459	CGG (R) => TGG (W)
ccmFn	1393	465	CGT (R) => TGT (C)
ccmFn	1436	479	TCG (S) => TTG (L)
ccmFn	1456	486	CTT (L) => TTT (F)
ccmFn	1460	487	CCA (P) => CTA (L)
ccmFn	1472	491	TCA (S) => TTA (L)
ccmFn	1481	494	TCT (S) => TTT (F)
ccmFn	1507	503	CCC (P) => TCC (S)
cob	118	40	CCG (P) => TCG (S)
cob	286	96	CTC (L) => TTC (F)
cob	298	100	CAC (H) => TAC (Y)
cob	325	109	CAT (H) => TAT (Y)
cob	358	120	CGG (R) => TGG (W)
cob	419	140	CCA (P) => CTA (L)
cob	568	190	CAT (H) => TAT (Y)
cob	680	227	TCT (S) => TTT (F)
cob	737	246	TCT (S) => TTT (F)
cob	808	270	CCC (P) => TCC (S)
cob	853	285	CAT (H) => TAT (Y)
cob	908	303	CCA (P) => CTA (L)
cob	982	328	CAC (H) => TAC (Y)
cob	1015	339	CGC (R) => TGC (C)

cob	1084	362	CCT (P) => TCT (S)
cox1	11	4	CCG (P) => CTG (L)
cox1	242	81	TCT (S) => TTT (F)
cox1	254	85	TCT (S) => TTT (F)
cox1	352	118	CCA (P) => TCA (S)
cox1	443	148	TCA (S) => TTA (L)
cox1	452	151	TCT (S) => TTT (F)
cox1	458	153	CCT (P) => CTT (L)
cox1	515	172	TCC (S) => TTC (F)
cox1	551	184	TCA (S) => TTA (L)
cox1	590	197	CCA (P) => CTA (L)
cox1	668	223	TCT (S) => TTT (F)
cox1	715	239	CGG (R) => TGG (W)
cox1	746	249	CCC (P) => CTC (L)
cox1	761	254	TCC (S) => TTC (F)
cox1	854	285	CCT (P) => CTT (L)
cox1	860	287	TCT (S) => TTT (F)
cox1	1037	346	TCC (S) => TTC (F)
cox1	1079	360	CCG (P) => CTG (L)
cox1	1186	396	CAC (H) => TAC (Y)
cox1	1274	425	CCG (P) => CTG (L)
cox1	1373	458	TCT (S) => TTT (F)
cox1	1405	469	CGT (R) => TGT (C)
cox1	1433	478	TCA (S) => TTA (L)
cox1	1489	497	CCA (P) => TCA (S)
cox1	1499	500	CCG (P) => CTG (L)
cox2	71	24	TCT (S) => TTT (F)
cox2	161	54	TCA (S) => TTA (L)
cox2	163	55	CGG (R) => TGG (W)
cox2	253	85	CGG (R) => TGG (W)
cox2	278	93	CCG (P) => CTG (L)
cox2	379	127	CGG (R) => TGG (W)
cox2	443	148	ACG (T) => ATG (M)
cox2	581	194	TCA (S) => TTA (L)
cox2	632	211	TCG (S) => TTG (L)
cox2	790	264	CAG (Q) => TAG (X)
cox3	245	82	CCT (P) => CTT (L)
cox3	289	97	CTT (L) => TTT (F)
cox3	298	100	CTT (L) => TTT (F)
cox3	304	102	CGG (R) => TGG (W)
cox3	311	104	TCT (S) => TTT (F)
cox3	314	105	TCT (S) => TTT (F)
cox3	388	130	CGG (R) => TGG (W)
cox3	419	140	CCC (P) => CTC (L)
cox3	512	171	TCA (S) => TTA (L)
cox3	566	189	TCC (S) => TTC (F)
cox3	653	218	TCG (S) => TTG (L)
cox3	754	252	CGG (R) => TGG (W)
cox3	764	255	CCA (P) => CTA (L)

matR	32	11	TCC (S) => TTC (F)
matR	193	65	CCC (P) => TCC (S)
matR	235	79	CCC (P) => TTC (F)
matR	236	79	CCC (P) => TTC (F)
matR	275	92	GCG (A) => GTG (V)
matR	326	109	CCA (P) => CTA (L)
matR	506	169	GCG (A) => GTG (V)
matR	1679	560	TCC (S) => TTC (F)
matR	1700	567	CCT (P) => CTT (L)
matR	1720	574	CGC (R) => TGC (C)
matR	1756	586	CAC (H) => TAC (Y)
matR	1787	596	CCG (P) => CTG (L)
matR	1826	609	CCA (P) => CTA (L)
matR	1844	615	TCA (S) => TTA (L)
mttB	35	12	CCG (P) => CTG (L)
mttB	73	25	CGG (R) => TGG (W)
mttB	109	37	CGT (R) => TGT (C)
mttB	121	41	CCG (P) => TCG (S)
mttB	137	46	TCT (S) => TTT (F)
mttB	140	47	CCA (P) => CTA (L)
mttB	193	65	CGT (R) => TGT (C)
mttB	203	68	TCA (S) => TTA (L)
mttB	215	72	TCC (S) => TTC (F)
mttB	217	73	CCG (P) => TCG (S)
mttB	277	93	CAT (H) => TAT (Y)
mttB	343	115	CTC (L) => TTC (F)
mttB	346	116	CAT (H) => TAT (Y)
mttB	359	120	TCT (S) => TTT (F)
mttB	361	121	CGC (R) => TGC (C)
mttB	368	123	TCC (S) => TTC (F)
mttB	389	130	CCT (P) => CTT (L)
mttB	391	131	CCC (P) => TCC (S)
mttB	394	132	CGG (R) => TGG (W)
mttB	422	141	CCA (P) => CTA (L)
mttB	452	151	TCG (S) => TTG (L)
mttB	487	163	CAT (H) => TAT (Y)
mttB	512	171	TCG (S) => TTG (L)
mttB	520	174	CCA (P) => TCA (S)
mttB	556	186	CGT (R) => TGT (C)
mttB	563	188	CCA (P) => CTA (L)
mttB	569	190	CCA (P) => CTA (L)
mttB	593	198	TCC (S) => TTC (F)
mttB	625	209	CCG (P) => TCG (S)
mttB	698	233	TCG (S) => TTG (L)
mttB	719	240	TCT (S) => TTT (F)
mttB	728	243	TCG (S) => TTG (L)
nad1	215	72	TCC (S) => TTC (F)
nad1	265	89	CGG (R) => TGG (W)
nad1	308	103	CCG (P) => CTG (L)

nad1	376	126	CGG (R) => TGG (W)
nad1	436	146	CCT (P) => TCT (S)
nad1	490	164	CCC (P) => TCC (S)
nad1	493	165	CGT (R) => TGT (C)
nad1	500	167	TCG (S) => TTG (L)
nad1	536	179	TCC (S) => TTC (F)
nad1	635	212	TCA (S) => TTA (L)
nad1	674	225	TCT (S) => TTT (F)
nad1	683	228	TCT (S) => TTT (F)
nad1	725	242	CCA (P) => CTA (L)
nad1	734	245	TCG (S) => TTG (L)
nad1	740	247	TCT (S) => TTT (F)
nad1	743	248	CCA (P) => CTA (L)
nad1	755	252	CCG (P) => CTG (L)
nad1	802	268	CGG (R) => TGG (W)
nad1	823	275	CTT (L) => TTT (F)
nad1	898	300	CGG (R) => TGG (W)
nad1	928	310	CGG (R) => TGG (W)
nad1	937	313	CCC (P) => TCC (S)
nad2	26	9	TCC (S) => TTC (F)
nad2	262	88	CTT (L) => TTT (F)
nad2	308	103	TCT (S) => TTT (F)
nad2	311	104	TCC (S) => TTC (F)
nad2	335	112	TCT (S) => TTT (F)
nad2	341	114	TCC (S) => TTC (F)
nad2	356	119	CCA (P) => CTA (L)
nad2	361	121	CCT (P) => TCT (S)
nad2	367	123	CGC (R) => TGC (C)
nad2	401	134	TCA (S) => TTA (L)
nad2	428	143	CCT (P) => CTT (L)
nad2	497	166	TCG (S) => TTG (L)
nad2	662	221	TCT (S) => TTT (F)
nad2	677	226	TCC (S) => TTC (F)
nad2	788	263	TCT (S) => TTT (F)
nad2	800	267	TCA (S) => TTA (L)
nad2	809	270	TCT (S) => TTT (F)
nad2	928	310	CAT (H) => TAT (Y)
nad2	958	320	CGT (R) => TGT (C)
nad2	962	321	ACT (T) => ATT (I)
nad2	1028	343	TCA (S) => TTA (L)
nad2	1058	353	TCA (S) => TTA (L)
nad2	1127	376	TCG (S) => TTG (L)
nad2	1247	416	CCA (P) => CTA (L)
nad2	1276	426	CGT (R) => TGT (C)
nad2	1400	467	TCA (S) => TTA (L)
nad2	1409	470	CCA (P) => CTA (L)
nad2	1457	486	TCA (S) => TTA (L)
nad3	5	2	TCA (S) => TTA (L)
nad3	44	15	CCG (P) => CTG (L)

nad3	62	21	CCA (P) => CTA (L)
nad3	80	27	CCA (P) => CTA (L)
nad3	208	70	CCT (P) => TTT (F)
nad3	209	70	CCT (P) => TTT (F)
nad3	215	72	CCG (P) => CTG (L)
nad3	230	77	TCC (S) => TTC (F)
nad3	247	83	CCT (P) => TCT (S)
nad3	251	84	CCC (P) => CTC (L)
nad3	266	89	CCC (P) => CTC (L)
nad3	275	92	TCT (S) => TTT (F)
nad3	317	106	TCT (S) => TTT (F)
nad3	344	115	TCG (S) => TTG (L)
nad3	349	117	CGG (R) => TGG (W)
nad4	29	10	TCT (S) => TTT (F)
nad4	74	25	ACT (T) => ATT (I)
nad4	77	26	CCT (P) => CTT (L)
nad4	107	36	CCG (P) => CTG (L)
nad4	158	53	CCT (P) => CTT (L)
nad4	166	56	CGG (R) => TGG (W)
nad4	197	66	TCT (S) => TTT (F)
nad4	362	121	ACA (T) => ATA (I)
nad4	368	123	TCT (S) => TTT (F)
nad4	376	126	CGT (R) => TGT (C)
nad4	403	135	CGC (R) => TGC (C)
nad4	416	139	CCT (P) => CTT (L)
nad4	433	145	CTT (L) => TTT (F)
nad4	436	146	CCC (P) => TTC (F)
nad4	437	146	CCC (P) => TTC (F)
nad4	517	173	CTT (L) => TTT (F)
nad4	1433	478	CCG (P) => CTG (L)
nad4L	11	4	TCT (S) => TTT (F)
nad4L	17	6	TCA (S) => TTA (L)
nad4L	25	9	CGG (R) => TGG (W)
nad4L	56	19	CCT (P) => CTT (L)
nad4L	65	22	TCA (S) => TTA (L)
nad4L	70	24	CCA (P) => TCA (S)
nad4L	80	27	TCA (S) => TTA (L)
nad4L	101	34	TCG (S) => TTG (L)
nad4L	128	43	TCG (S) => TTG (L)
nad4L	149	50	TCA (S) => TTA (L)
nad4L	158	53	TCA (S) => TTA (L)
nad4L	167	56	CCA (P) => CTA (L)
nad4L	251	84	TCT (S) => TTT (F)
nad5	155	52	CCG (P) => CTG (L)
nad5	242	81	CCG (P) => CTG (L)
nad5	272	91	TCC (S) => TTC (F)
nad5	358	120	CCT (P) => TTT (F)
nad5	359	120	CCT (P) => TTT (F)
nad5	374	125	CCA (P) => CTA (L)

nad5	398	133	TCT (S) => TTT (F)
nad5	539	180	CCT (P) => CTT (L)
nad5	548	183	TCG (S) => TTG (L)
nad5	629	210	TCT (S) => TTT (F)
nad5	631	211	CGC (R) => TGC (C)
nad5	676	226	CTT (L) => TTT (F)
nad5	689	230	GCT (A) => GTT (V)
nad5	713	238	TCG (S) => TTG (L)
nad5	725	242	TCA (S) => TTA (L)
nad5	835	279	CCA (P) => TCA (S)
nad5	1184	395	CCA (P) => CTA (L)
nad5	1310	437	TCA (S) => TTA (L)
nad5	1490	497	CCC (P) => CTC (L)
nad5	1550	517	ACC (T) => ATC (I)
nad5	1568	523	CCG (P) => CTG (L)
nad5	1580	527	TCA (S) => TTA (L)
nad5	1589	530	TCT (S) => TTT (F)
nad5	1610	537	CCA (P) => CTA (L)
nad5	1895	632	TCA (S) => TTA (L)
nad5	1916	639	TCT (S) => TTT (F)
nad5	1918	640	CGT (R) => TGT (C)
nad5	1958	653	TCG (S) => TTG (L)
nad6	7	3	CTT (L) => TTT (F)
nad6	88	30	CCC (P) => TTC (F)
nad6	89	30	CCC (P) => TTC (F)
nad6	95	32	CCA (P) => CTA (L)
nad6	103	35	CGC (R) => TGC (C)
nad6	161	54	CCA (P) => CTA (L)
nad6	169	57	CAT (H) => TAT (Y)
nad6	191	64	TCA (S) => TTA (L)
nad6	446	149	TCC (S) => TTC (F)
nad6	463	155	CCT (P) => TCT (S)
nad6	569	190	TCT (S) => TTT (F)
nad7	38	13	TCG (S) => TTG (L)
nad7	77	26	TCA (S) => TTA (L)
nad7	83	28	TCA (S) => TTA (L)
nad7	137	46	TCA (S) => TTA (L)
nad7	200	67	TCT (S) => TTT (F)
nad7	209	70	TCA (S) => TTA (L)
nad7	224	75	ACG (T) => ATG (M)
nad7	244	82	CAT (H) => TAT (Y)
nad7	251	84	TCA (S) => TTA (L)
nad7	316	106	CGT (R) => TGT (C)
nad7	335	112	TCA (S) => TTA (L)
nad7	344	115	TCA (S) => TTA (L)
nad7	383	128	TCA (S) => TTA (L)
nad7	533	178	TCC (S) => TTC (F)
nad7	578	193	TCA (S) => TTA (L)
nad7	679	227	CCA (P) => TCA (S)

nad7	698	233	TCG (S) => TTG (L)
nad7	724	242	CAT (H) => TAT (Y)
nad7	734	245	TCG (S) => TTG (L)
nad7	739	247	CCT (P) => TTT (F)
nad7	740	247	CCT (P) => TTT (F)
nad7	769	257	CGC (R) => TGC (C)
nad7	836	279	CCT (P) => CTT (L)
nad7	926	309	TCA (S) => TTA (L)
nad7	944	315	CCT (P) => CTT (L)
nad7	973	325	CCT (P) => TCT (S)
nad7	1057	353	CGT (R) => TGT (C)
nad7	1079	360	TCT (S) => TTT (F)
nad7	1088	363	TCA (S) => TTA (L)
nad7	1103	368	TCT (S) => TTT (F)
nad7	1124	375	CCA (P) => CTA (L)
nad7	1166	389	TCT (S) => TTT (F)
nad9	113	38	CCA (P) => CTA (L)
nad9	167	56	TCG (S) => TTG (L)
nad9	190	64	CAT (H) => TAT (Y)
nad9	298	100	CCG (P) => TCG (S)
nad9	311	104	CCA (P) => CTA (L)
nad9	328	110	CGG (R) => TGG (W)
nad9	368	123	TCC (S) => TTC (F)
nad9	398	133	TCA (S) => TTA (L)
nad9	439	147	CTT (L) => TTT (F)
nad9	539	180	TCT (S) => TTT (F)
rpl10	101	34	TCA (S) => TTA (L)
rpl10	134	45	CCA (P) => CTA (L)
rpl10	239	80	TCG (S) => TTG (L)
rpl16	37	13	CAG (Q) => TAG (X)
rpl16	107	36	TCT (S) => TTT (F)
rpl16	185	62	ACT (T) => ATT (I)
rpl16	313	105	CTC (L) => TTC (F)
rpl5	35	12	TCA (S) => TTA (L)
rpl5	47	16	CCG (P) => CTG (L)
rpl5	59	20	CCG (P) => CTG (L)
rpl5	64	22	CAC (H) => TAC (Y)
rpl5	92	31	TCG (S) => TTG (L)
rpl5	170	57	CCG (P) => CTG (L)
rpl5	518	173	CCA (P) => CTA (L)
rpl5	521	174	CCG (P) => CTG (L)
rps12	71	24	TCG (S) => TTG (L)
rps12	100	34	CGC (R) => TGC (C)
rps12	104	35	CCG (P) => CTG (L)
rps12	112	38	CCA (P) => TCA (S)
rps12	146	49	CCA (P) => CTA (L)
rps12	196	66	CAC (H) => TAC (Y)
rps12	221	74	TCG (S) => TTG (L)
rps12	269	90	TCG (S) => TTG (L)

rps12	284	95	TCC (S) => TTC (F)
rps13	26	9	TCA (S) => TTA (L)
rps13	56	19	TCA (S) => TTA (L)
rps13	100	34	CGT (R) => TGT (C)
rps13	287	96	TCA (S) => TTA (L)
rps3	64	22	CGG (R) => TGG (W)
rps3	92	31	TCA (S) => TTA (L)
rps3	397	133	CCG (P) => TCG (S)
rps3	512	171	TCA (S) => TTA (L)
rps3	574	192	CTC (L) => TTC (F)
rps3	671	224	CCA (P) => CTA (L)
rps3	716	239	TCG (S) => TTG (L)
rps3	956	319	CCT (P) => CTT (L)
rps3	1025	342	CCA (P) => CTA (L)
rps3	1382	461	CCG (P) => CTG (L)
rps3	1409	470	CCG (P) => CTG (L)
rps3	1520	507	TCA (S) => TTA (L)
rps3	1606	536	CCT (P) => TCT (S)
rps3	1637	546	TCA (S) => TTA (L)
rps4	184	62	CCC (P) => TCC (S)
rps4	193	65	CAT (H) => TAT (Y)
rps4	257	86	CCA (P) => CTA (L)
rps4	266	89	CCA (P) => CTA (L)
rps4	278	93	TCG (S) => TTG (L)
rps4	290	97	CCG (P) => CTG (L)
rps4	335	112	CCG (P) => CTG (L)
rps4	479	160	TCA (S) => TTA (L)
rps4	947	316	TCT (S) => TTT (F)
rps4	995	332	TCG (S) => TTG (L)
rps4	1006	336	CAT (H) => TAT (Y)
rps4	1016	339	CCA (P) => CTA (L)
rps4	1031	344	TCT (S) => TTT (F)
sdh4	155	52	CCA (P) => CTA (L)
sdh4	203	68	CCA (P) => CTA (L)
sdh4	259	87	CAT (H) => TAT (Y)
sdh4	353	118	TCT (S) => TTT (F)
